# Unsupervised Classification of Surface Defects in Wire Rod Production Obtained by Eddy Current Sensors

**DOI:** 10.3390/s150510100

**Published:** 2015-04-29

**Authors:** Sergio Saludes-Rodil, Enrique Baeyens, Carlos P. Rodríguez-Juan

**Affiliations:** 1 Centro Tecnológico CARTIF, Parque Tecnológico de Boecillo 205, 47151 Boecillo, Valladolid, Spain; 2 Instituto de las Tecnologías Avanzadas de la Producción, Universidad de Valladolid, Paseo del cauce 59, 47011 Valladolid, Spain; E-Mail: enrbae@eis.uva.es; 3 ISEND S.A., Parque Tecnológico de Boecillo, Luis Proust 10, 47151 Boecillo, Valladolid, Spain; E-Mail: carlos@isend.es

**Keywords:** dynamic time warping, cluster analysis, modified Fourier descriptors, unsupervised classification, wire rod manufacturing, eddy current inspection, nondestructive testing

## Abstract

An unsupervised approach to classify surface defects in wire rod manufacturing is developed in this paper. The defects are extracted from an eddy current signal and classified using a clustering technique that uses the dynamic time warping distance as the dissimilarity measure. The new approach has been successfully tested using industrial data. It is shown that it outperforms other classification alternatives, such as the modified Fourier descriptors.

## 1. Introduction

Wire rods made using the hot rolling technique can present surface defects. Several techniques have been applied to detect the surface defects that appear during wire rod manufacturing. Approaches based on image processing have been proposed in [1,2]. Alternatively to computer vision-based techniques, the eddy current nondestructive technique is effectively used to detect surface defects [3]. The basic instrument for eddy current inspection is a coil fed with an alternating electric current. The complex impedance of the coil *Z*_0_ changes in accordance with the eddy current redistribution due to material defects or inhomogeneities [4,5].

Besides detection, defect classification is of industrial interest, and a great deal of research has been devoted to this issue. Many approaches are based on signal processing or shape characterization followed by a supervised classifier. This implies the use of labeled defect sets covering all possible defect types. A complete knowledge base that includes as many examples as possible of every type of possible defect is crucial to develop a good classification procedure. This is a serious drawback, because it is not always easy nor even possible in industrial practice to collect a number of examples large enough to build a useful knowledge base.

There is an interest in developing automated eddy current-based inspection systems able to detect and classify defects. However, the lack of adequate defect collections in hot rolling industrial plants prevents the design of supervised defect classifiers. Motivated by this fact, we proposed an unsupervised classifier that can aid the plant operators to build their knowledge bases and classify and analyze the surface defects appearing in their products.

The rest of the paper is organized as follows. In Section 2, the problem of the classification of surface defects obtained by eddy current supervision in the manufacturing of a wire rod is described and formulated. A revision of related work and the main contributions in our work are also included. In Section 3, several methods of unsupervised classification are explained, and the normalized dynamic time warping distance that will be used as a dissimilarity metric between defect sequences is also introduced. The results of an experiment using real data obtained during the production of the wire rod are reported in Section 4. Section 5 contains the discussion of the results. Finally, some conclusions are given in Section 6.

## 2. Description of the Problem

### 2.1. Eddy Current Inspection in Wire Rod Manufacturing

Wire rods are an intermediate steel product of approximately a round solid cross section that is wound into coils and transported in this form. It is primarily used for subsequent drawing and finishing by wire drawers and is ultimately used to manufacture a variety of products, including electric welded chains, cold-drawn bars, springs, nails, reinforcing wire mesh, chain link fence and many different types of wires. Bar and wire rods are produced by hot rolling, and surface defects can appear on rods during the manufacturing process. These defects can be detected by an eddy current inspection system.

**Figure 1:**
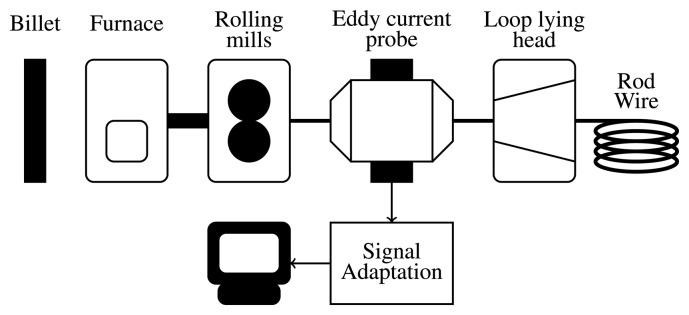
Layout of a wire rod mill with the eddy current inspection system.

The layout of a wire rod mill and its inspection system are schematically represented in Figure 1. The eddy current probe is placed at the end of the mill process and operates when the wire is still hot, at around 927 °C, depending on the material.

The probe used belongs to the differential class and was managed by an ISEND HOTanalyzer system. The authors would like to not disclose the operation details due to confidentiality reasons. The methods presented in this paper are independent of the probe operational parameters.

The eddy current inspection system directly acquires impedance measurements from the product line. It isolates the recorded parts where the impedance of the coil probe experiences a change that corresponds to a surface defect.

The inspection signal is a sequence of measurements of the complex impedance:

(1) $$Z_{0}\left(t\right)=x\left(t\right)+jy\left(t\right)$$

A surface defect is a finite subsequence of the complex impedance signal *Z*_0_(*t*).

The industrial supervision system of Figure 1 is continuously collecting measurements and produces a large amount of data that are not possible to analyze by a human operator without the aid of some automatic computer system. Our solution is motivated by the requirements of the operators in a real manufacturing plant and consists of an automatic system that analyzes the eddy current signal recorded during a production batch and extracts every subsequence of interest where the impedance changes. These subsequences are the collection Σ of surface defects to be classified.

The defect collection Σ is classified offline using unsupervised classification methods, and the results are provided to the operators for their posterior analysis. Hence, our solution avoids a very tedious, unpractical and almost unfeasible human classification process and can be considered as an initial step towards an online unsupervised classification system.

### 2.2. Problem Formulation

In order to formalize the problem, the impedance measurement at time *t* is assumed to belong to a metric space ( 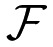, *d*) where 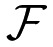 is the underlying set and *d* is the distance on that set. In our case, the set 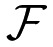 can be either the complex plane ℂ or the Euclidean plane R^2^, which are mathematically equivalent. Let 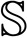
( 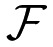) denote the set of every finite sequence on 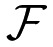. Hence, any defect is modeled as an element of 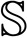
( 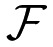), and the collection of defects is given by:

(2) $$\sum =\left\{{\text{x}}_{k}\in \text{S}\left(F\right):k=1,\ldots ,K\right\}$$

The problem of interest can be posed as follows: given a collection Σ of unlabeled defects corresponding to an unknown number *K* of defect types, determine the number *K* and find a partition {Σ*_k_* : *k* = 1,…, *N*}, such that each defect in the subset Σ*_k_* belongs to the same defect type. A partition {Σ*_k_* : *k* = 1,…, *K*} of Σ satisfies that Σ*_k_* ⊂ Σ for any *k*, Σ*_j_* ∩ Σ*_k_* = 0 for *j* ≠ *k* and $\cup_{k=1}^{K}\sum_{k}=\sum$.

The main difficulties in this problem are that the defects are unlabeled, and the different types of defects are unknown in advance. Besides, the sequences representing individual defects have different lengths, even for defects belonging to the same class. The features characterizing different defect classes are related to the shape and orientation of the polar graph of the complex impedance, but they are independent of the length and scale. These difficulties motivate the use of unsupervised classification methods and the definition of a metric for sequences of different lengths that allows the right classification of defects. The unsupervised classification methods considered are the *K*-medoids clustering algorithm and the evolving self-organizing map algorithm. As a metric for the dissimilarity of defect sequences, the normalized dynamic time warping distance is used.

### 2.3. Related Work

Classification algorithms for eddy current testing can be arranged into two main categories:

1. Signal processing based: Eddy current signals are processed in order to extract some characteristics from them, allowing differentiating among defect types, as in [6–11].
2. Shape based: The shape of the eddy current signals in the impedance plane are processed to find out the contours or appearances associated with every type of defect, as in [12–15].

Signal processing-based techniques are the most commonly found in the literature. Every technique in this class processes the eddy current signals in order to obtain a finite set of numeric values that unequivocally characterizes every type of defect.

Time-frequency transformation, like wavelet analysis, has been extensively used to process eddy current signals. In [6], several applications to detect defects in nuclear power generation components are reported. In [7], the wavelet analysis is used to enhance the eddy current signals prior to defect detection. Time domain methods have also been proposed [8]. The Hilbert transform [9] and the principal component analysis [10] are some of the techniques used to extract features from the eddy current signals. Furthermore, neural networks have been applied in [11].

Contrarily to signal processing based methods, there are other methods that rely on the shape that the impedance takes in the complex plane. Most of these methods are based on the modified Fourier descriptors [16], which are used to describe closed curves by a finite set of numerical features. This technique is briefly described in Section 3. Modified Fourier descriptors have been applied to classify eddy current signals in [12–15].

### 2.4. Main Contributions

The main contribution in this paper is an efficient unsupervised method for classifying surface defects in the manufacturing of wire rods using eddy current inspection. This method comprises two key elements. First, a new defect dissimilarity measure for eddy current signals is introduced. This measure uses the normalized dynamic time warping (DTW). Second, a clustering approach that uses the DTW distance is applied in an unsupervised way. The *K*-medoids clustering algorithm has been successfully tested. In addition, an evolving self-organizing map (ESOM) has been applied to obtain a set of defect prototypes that are later classified using the *K*-medoids clustering algorithm. A defect is classified by the cluster corresponding to the closest prototype in the DTW distance. The ESOM also uses the normalized DTW measure, and its goal is to obtain a parsimonious representation of the defects collection Σ that can be preserved from a production shift to the next one. The ESOM is evolving with any new defect, but the clustering process is accomplished only once for each shift. The techniques used in this paper are not new; however, to the knowledge of the authors, they have not been previously used in conjunction to classify surface defects in wire rod manufacturing. The resultant approach has been demonstrated to be very effective and outperforms other alternatives based on modified Fourier descriptors that have been extensively used in feature extraction of signal obtained by eddy current inspection.

## 3. Methods

### 3.1. Modified Fourier Descriptors

Let **x** ∈ 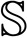
(ℂ) be a finite sequence of complex numbers representing the impedances corresponding to a surface defect. Let *N* = |**x**| be the length of the sequence, then:

(3) $$\text{x}=\left\{x_{k}:k=1,\ldots ,N\right\}$$

The sequence **x** can be equivalently represented by the Fourier descriptors $\left\{f_{k}:k=-\frac{N}{2}+1,\ldots ,\frac{N}{2}\right\}$, which are the coefficients of the Fourier transform of **x**:

(4) $$x_{k}=\sum_{\ell =-\frac{N}{2}+1}^{\frac{N}{2}}f_{k}{\text{e}}^{j2\pi k\ell /N}$$

with:

(5) $$f_{k}=\frac{1}{N}\sum_{\ell =0}^{N}x_{k}{\text{e}}^{-\text{j}2\pi k\ell /N}$$

The defect shape in the impedance plane is completely described by the Fourier descriptors *f_k_*. However, they are sensitive to signal transformations, such as translation, scale change and reverse description.

An alternative description is proposed in [16] to overcome this drawback. It consists of using nonlinear combinations of the Fourier descriptors:

(6) $$b_{k}=\frac{f_{1+k}\;f_{1-k}}{f_{1}^{2}}\;\text{with}\;k=2,3,\ldots ,\frac{N}{2}-1$$

(7) $$b_{1}=\frac{f_{2}\left|f_{1}\right|}{f_{1}^{2}}$$

These are the Grandlun's modified Fourier descriptors. They contain information about shape and are invariant under translation and scale change. Only *b*_1_ is sensitive to rotation, which provides information about the overall defect phase.

The main disadvantage of Grandlun's modified Fourier descriptors is that they are affected by reverse description, *i.e.*, their value depends on the direction that the defect passes through the probe. To avoid this problem, a modified formulation is proposed in [13]:

(8) $$d_{k}=\frac{f_{k}f_{-k}}{\left|f_{1}f_{-1}\right|}\;\text{with}\;k=1,2,\ldots ,\frac{N}{2}-1$$

The Oukhellou modified Fourier descriptors contain information about the shape of the defect and are invariant under translation, scale change and reverse description. Besides, they are also sensitive to rotation changes, so they provide information about the defect phase.

### 3.2. Dynamic Time Warping

Dynamic time warping (DTW) [17,18] is a well-known technique to obtain the optimal alignment between two given time-dependent sequences under certain restrictions. Intuitively, the sequences are warped in a nonlinear fashion to match each other.

Let **x**,**y** ∈ 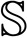
(ℝ^2^) be two sequences of length *N* = |**x**| and *M* = |**y**|, respectively, where *d* is the Euclidean distance in ℝ^2^. In order to align these sequences using DTW, a matrix *N*-by-*M* is constructed. The element (*i*,*j*) of this matrix contains the Euclidean distance *d* (*x_i_*, *y_j_*) between the two points *x_i_* ∈ **x** and *y_j_* ∈ **y**. A warping path **w** is a finite sequence of *K* pairs of natural numbers **w** ≔ {*w_k_* ∈ ℕ × ℕ: *k* = 1,2,…, *K*} satisfying the following conditions:

1. Path length: the length of the warping path is bounded by :
  (9) max{N,M}≤L≤N+M−1
2. Boundary condition: the initial and final values of the warping path are given by :
  (10) $$\begin{array}{ll} w_{1}=\left(1,1\right), & w_{L}=\left(N,M\right) \end{array}$$
3. Step size condition: the warping path cannot increase more than one in each dimension :
  (11) $$\begin{array}{r} w_{k+1}-w_{k}\in \left\{\left(0,1\right),\left(1,0\right),\left(1,1\right)\right\}\\ k=1,\ldots ,L-1 \end{array}$$

Let 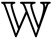
(**x**, **y**) denote the set of all possible warping paths for two finite sequences **x** and **y** of elements of the set 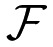. The distance *D* (**w**; **x**, **y**) of the sequences **x** and **y** with respect to the warping path **w** ∈ 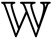
(**x**, **y**) is defined as:

(12) $$D\left(\text{w};\text{x},\text{y}\right):=\sum_{\ell =1}^{\left|\text{w}\right|}d(x_{w_{\ell ,1}},y_{w_{\ell ,2}})$$

Furthermore, an optimal warping path for the sequences **x** and **y** is a warping path w* ∈ 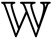
(**x**, **y**) having minimal distance for those sequences with respect to all possible warping paths. The DTW distance D*(**x**, **y**) between the sequences **x** and **y** is then defined as the distance of those sequences with respect to an optimal warping path:

(13) $$D^{*}\left(\text{x},\text{y}\right)=\min\;\left\{D\left(\text{w};\text{x},\text{y}\right):\text{w}\in \text{W}\left(\text{x},\text{y}\right)\right\}$$

The optimal path is computed by applying dynamic programming to Equation (12) that defines the distance with respect to the warping path.

The DTW distance is sensitive to the length of the sequences. Since the DTW distance is usually applied to sequences of different lengths, it can be normalized dividing by the length of the optimal warping path. The normalized DTW distance between two finite length sequences **x**,**y** ∈ 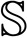
( 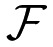) is defined as:

(14) $$\Delta \left(\text{x},\text{y}\right)=K^{-1}D^{*}\left(\text{x},\text{y}\right)$$

where *K* is the length of the optimal warping path, *i.e.*, *K* = |**w**|.

An algorithm that computes the normalized DTW distance is given in Algorithm 1.



**Algorithm 1.** Normalized DTW distance.
Let **x**, **y** ∈ 

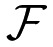
 be two finite sequences of lengths *N* = |**x**| and *M* = |**y**| , respectively. The normalized DTW distance Δ(**x**, **y**) is computed as follows:1.Initialize *δ*_(0,0)_ = 0, *δ*_(_*_n_*_,0)_ = ∞ for *n* = 1,…, *N* and *δ*_(0,_*_m_*_)_ = ∞ for *m* = 1,…, *M* and compute the terms of the DTW matrix, using the difference equation:   *δ*_(_*_n_*_,_
*_m_*_)_ = min {*δ*_(_*_n_*_−1,_*_m_*_)_, *δ*_(_*_n_*_,_*_m_*_−1)_, *δ*_(_*_n_*_−1,_*_m_*_−1)_} + *d*(*x_n_*, *x_m_*)for (*n*, *m*) ∈ {1,…, *N*} × {1,…, *M*}.2.Initialize *ℓ* = 0, *v_ℓ_* = (*N*, *M*) and compute the sequence **v** ∈ ℕ^2^ as follows: while [*v_ℓ_* ≠ (1,1)], repeat:   *ℓ* = *ℓ* + 1,  *v_ℓ_* ∈ argmin{*δ_v_* : *v* ∈ {*v_ℓ_*_−1_ − (1,0), *v_ℓ_*_−1_ − (0,1), *v_ℓ_*_−1_ − (1,1)}} end while loopthen *K* = |**v**| and w* = {*w_ℓ_* : w*_ℓ_* = *s_K_*_−_*_ℓ_*_+1_(**v**)}.3.The normalized DTW distance between the sequences **x** and **y** is Δ(**x**, **y**) = *k*^−1^*δ*_(_*_N_*_,_*_M_*_)_.


### 3.3. The K-Medoids Algorithm

Clustering methods are used to classify a collection of objects Σ into different classes without human intervention. A well-known hard clustering method is the given by the *K*-medoids algorithm [19]. Each cluster is represented by a vector selected among the elements Σ, which is a set of sequences to be classified into *K* groups. The representative element of each class is called a medoid. Apart form its medoid, each cluster contains all sequences in Σ that are not used as medoids in other clusters and lie closer to its medoid than to the medoids representing the other clusters. An algorithm to perform K-medoids clustering is given in Algorithm 2.

### 3.4. The Evolving Self-Organizing Map

The evolving self-organizing map (ESOM) [20,21] is used to obtain a parsimonious representation of a given set of elements Σ in terms of a reduced number of prototype elements and certain relationships between them. The ESOM is an evolving version of the self-organizing map (SOM). The main differences are that no topological constraint is given *a priori* for the feature map and that prototype elements are not organized onto a lattice. The ESOM is represented by a graph, where each prototype element is a node or vertex, and the relationships are represented by edges of different weights. The ESOM provides a preserving topology representation of the input space in terms of a reduced number of defect prototypes. This representation contains the relevant information about the defect classes that is preserved among production shifts.



**Algorithm 2.**
*K*-medoids algorithm.
Let Δ(**x**, **y**) denote the distance between two elements **x**, **y** ∈ Σ.1.Choose an arbitrary partition 

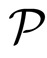
*_k_* = {Σ*_k_* : *k* = 1,…, *K*} of Σ and an arbitrary set of medoids Λ = {*m*(Σ*_k_*) ∈ Σ_k_ : *k* = 1,…, *K*}.2.For every Σ*_k_* ∈ 

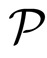
*_K_*, compute the elements that are wrongly classified, *i.e.*, **x** ∈ Σ*_k_* satisfying Δ(**x**, *m*(Σ*_j_*)) < Δ(**x**, *m*(Σ*_k_*)) for Σ*_j_* ≠ Σ*_k_*. For these elements, update the partition as follows:
$\sum_{k}^{\prime }=\sum_{k}-\left\{\text{x}\right\}$ and 
$\sum_{j}^{\prime }=\sum_{j}+\left\{\text{x}\right\}$. The resultant partition is 
${\text{P}}_{K}^{\prime }=\left\{\sum_{k}^{\prime }:k=1,\ldots ,K\right\}$.3.Obtain the medoids set 
$\wedge^{\prime }=\left\{\text{m}\left(\sum_{k}^{\prime }\right):\sum_{k}^{\prime }\in {\text{P}}_{K}^{\prime }\right\}$ for the new partition 
${\text{P}}_{K}^{\prime }$ by solving the following *K* optimization programs: 
$m\left(\sum_{k}^{\prime }\right)\in \arg\min\left\{\max\left\{\gamma_{k}:\Delta \left(\text{x},\text{y}\right)\leq \gamma_{k},\text{y}\in \sum_{k}^{\prime }\right\}:\text{x}\in \sum_{k}^{\prime }\right\},\sum_{k}^{\prime }\in {\text{P}}_{K}^{\prime }$4.If the medoids set does not change, *i.e.*, if Λ′ = Λ, then the clustering process is completed. Otherwise, do Λ = Λ′, and go to Step 2.


The ESOM network starts without any vertex. During learning, the network is updated to capture the on-line incoming data, creating new nodes and

edges when necessary. Edges are used to maintain the neighborhood relationships between close nodes. The connection strength is determined by the distance between connected nodes. If the distance is large, the edge weight is weak and it can be disregarded. In this way, the feature map can be split apart, and data structures, such as clusters and outliers can emerge.

The ESOM network is characterized by a triplet:

(15) N=(V,E,s)

where 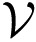 ⊂ 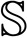
(ℝ^2^) is the vertex set containing the prototype nodes, 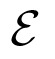 ⊂ 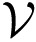 × 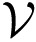 is the edge set and *s* : 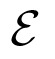 → ℝ is a function that provides the edge weights. For a set of defects Σ, the ESOM is obtained by applying an iterative algorithm with a set of parameters 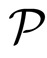 = {*ε*, *σ, γ*, *τ*}. The parameter *ϵ* controls the distance between different prototypes; *γ* is the learning rate; *σ* controls the spread of neighborhood; and *τ* is used for the preservation of the weakest connections. Usually *σ* = *ϵ* [20]. The learning process can be summarized in Algorithm .



**Algorithm 3.** Evolving self-organizing map.
1.Start with *k* = 0, 

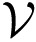
 = ∅, 

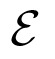
 = ∅.2.Choose a new **x** ∈ Σ and compute:
(16)$$V_{m}\left(\text{x}\right)=\left\{\text{y}\in V:\Delta \left(\text{x},\text{y}\right)<\epsilon \right\}$$If 

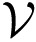
*_m_*(**x**) = ∅ go to 4.3.Update:
(17)$$V^{\prime }=V\cup \left\{\text{x}\right\}$$
(18)$$E^{\prime }=E\cup \left\{\left(\text{x},{\text{y}}_{1}\right),\left(\text{x},{\text{y}}_{2}\right)\right\}$$where:
(19)$$\Delta \left(\text{x},{\text{y}}_{1}\right)=\min\left\{\text{y}\;:\;\text{y}\in V\right\}$$
(20)$\Delta \left(\text{x},{\text{y}}_{2}\right)=\min\left\{\text{y}\;:\;\text{y}\neq {\text{y}}_{1},\text{y}\in V\right\}$and go to 5.4.Let y* be such that:
(21)$$\Delta \left(\text{x},{\text{y}}^{*}\right)=\min\left\{\Delta \left(\text{x},\text{y}\right):\;\text{y}\;\in V_{m}\right\}$$and 

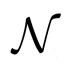
(y*) = {**y** : (**y**,y*) ∈ 

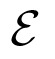
}. Update:
(22)$${\text{V}}^{\prime }=\left\{\phi \left(\text{y}\right):\;\text{y}\in V\right\}$$Where:
(23)$$\phi \left(\text{y}\right)=\{\begin{array}{ll} \left(1-\alpha \right)\text{y}+\alpha \text{x} & \text{y}\;\in \left\{{\text{y}}^{*}\cup N\left({\text{y}}^{*}\right)\right\}\\ \text{y} & \text{otherwise} \end{array}$$and:
(24)$\alpha =\gamma e^{-{\left|\Delta \left(\text{z},\text{x}\right)\right|}^{2}/2\sigma^{2}}$5.Update the connection strengths as follows:
(25)$$s\left({\text{y}}_{i},{\text{y}}_{j}\right)=\epsilon /\Delta \left({\text{y}}_{i},{\text{y}}_{j}\right)$$for any (**y***_i_*, **y***_j_*) ∈ 

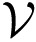
*′* × 

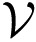
*′* and *i* ≠ *j*.6.Do *k′* = *k* + 1; if mod (*k′*, *τ*) = 0, remove the weakest connection.7.Do *k* = *k′*, 

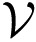
 = 

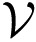
 and 

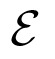
 = 

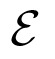
, and go to 2.


The distance between sequences Δ(**x**, **y**) is obtained using the DTW. Besides, since the sequences have different lengths, the sum operation in Equation (23) is not trivial, but it can be computed using the warping path. If **x** and **y** are sequences in 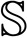
(ℝ^2^) with DTW distance Δ(**x**, **y**) and warping path w of length *L*, then the sum sequence **z** = **x** + **y** is a sequence of length *L*:

(26) $$\text{z}=\left\{z_{i}:i=1,\ldots ,L\right\}$$

where:

(27) $$\begin{array}{ll} z_{i}=x_{w_{1,i}}+y_{w_{2,i}}, & i=1,\ldots ,L \end{array}$$

The ESOM learning process is continuous and lasts indefinitely, so strict convergence of the algorithm is not a critical issue.

Clustering with the ESOM is accomplished over the prototype defects contained in the vertex set 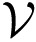. The *K*-medoids clustering algorithm can also be applied.

## 4. Experimental Results

### 4.1. Data Description

**Figure 2:**
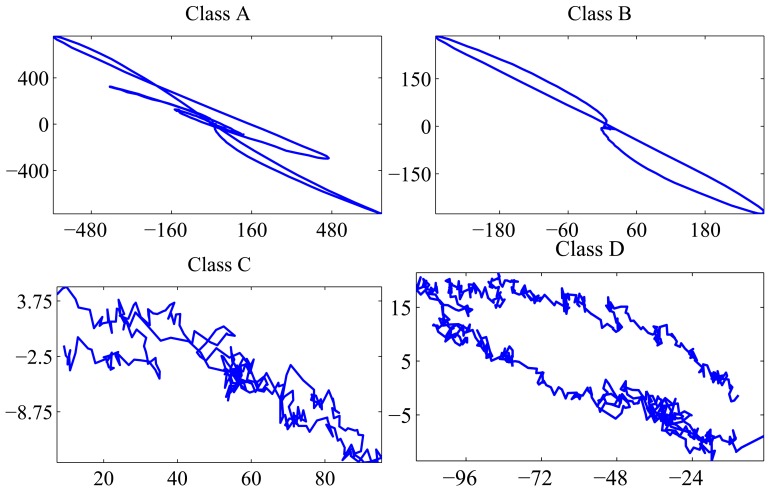
Individual defects belonging to each class represented in the complex impedance plane.

**Figure 3:**
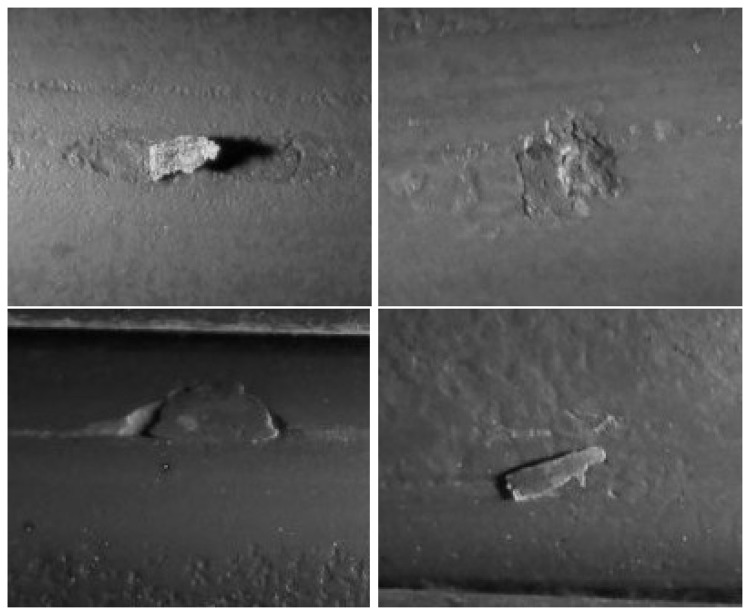
Macro photography of individual defects belonging to each class.

The operators of a manufacturing plant of wire rods identified and labeled the surface defects obtained for several production shifts. This has been a very tedious and time-consuming task, because it required unwinding long wire rod coils, searching the surface defects by visual inspection and classifying and putting them in correspondence with the signal recorded by the eddy current inspection system. After this manual process, a collection of labeled defects is available for validation of the developed unsupervised classification method. The surface defects have been classified by the experts into four different classes. The corresponding eddy current signals associated with them have been represented in the complex impedance plane and labeled as defects belonging to Classes A, B, C and D, respectively. An individual sequence representing each of these groups is depicted in Figure 2. The length of the available labeled sequences ranges between 101 and 996 samples. Samples of the defect classes are shown in Figure 3.

From a morphological viewpoint, Classes A and B feature lobes spreading across the second and fourth quadrants. Defects in Class A have more than two lobes, while defects in Class B exhibit exactly two lobes. Defects belonging to Classes C and D have only one lobe. The lobe of Class C defects elongates along the right side of the impedance plane, while the lobe of Class D defects goes through the left one. In our testing experiment, there are mime defects of Type A, 51 of Type B, 19 of Type C and 16 of Type D.

Two different approaches have been applied to this classification problem. Both of them are unsupervised classification approaches, as an alternative to the supervised approaches found in the literature; see Section 2. One of the approaches is based on the MFDand the other on the normalized DTW distance. Our results demonstrate that the method based on the DTW distance outperforms that based on MFD for this application.

### 4.2. Unsupervised Classification with Modified Fourier Descriptors

Unsupervised classification using the MFD is accomplished in two steps. The first one consists of computing the MDF for every defect according to Equation (8). In the second step, a clustering algorithm is applied to the MFD obtained in the first step.

The MFDs have been preprocessed through principal component analysis (PCA). The MFDs until the order 30 were computed, and the PCA analysis revealed that the two first principal components retained 99.63% of the variance. Several clustering algorithms were tried, but only spectral clustering [22] and ESOM-based clustering produced satisfactory results.

**Figure 4:**
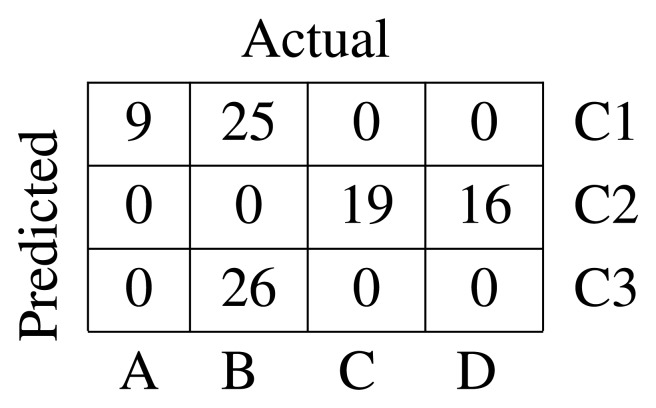
Confusion matrix for MFD and spectral clustering.

The adjacency matrix used as the starting point for spectral clustering has been computed over a *k* nearest neighbors (*k*-NN) similarity graph [22] with *k* = 15. The algorithm used is the normalized version, and the confusion matrix is presented in Figure 4. The silhouette index [19] is 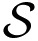 = 0.321. The silhouette index is a quantitative method of evaluating the results of a clustering process. It was proposed by Russeeuw in [23]. The confusion matrix shows that this method is not capable of discriminating defects in Classes C and D. Moreover, Class B is split into two different clusters, and one of the sequences is mixed with Class A, which is rightly assigned to a cluster.

The ESOM parameters are selected as *σ* = *ϵ* = 0.025, *γ* = 0.05 and *τ* = 10. The most critical parameter is *ϵ* and was found empirically, while *γ* has a small influence. The clustering method is based on computing the minimum spanning tree of the graph shaped by the prototypes and their connections. Prototypes in the same cluster are those that remain linked when inconsistent edges in the minimum spanning tree are removed. An edge is inconsistent when its weight is at least twice the mean of the weights associated with the other edges. The amount of edges averaged is chosen to maximize the silhouette index. This method can be considered as a gestalt clustering approach [24]. The original defects are clustered according to the closest prototype.

**Figure 5:**
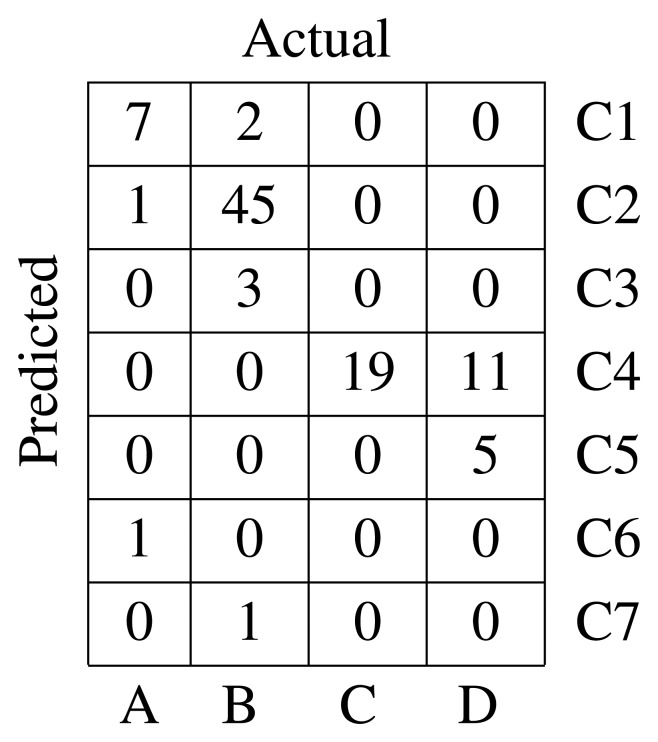
Confusion matrix for MFD and ESOM-based clustering.

The confusion matrix shown in Figure 5 summarizes the results. Seven clusters have been found, but two of them are negligible, because they contain only one element. Defects in Classes A and B are mainly assigned to Clusters C1 and C2, respectively. Defects in Class C are assigned to Cluster C4. Most of the defects in Class D are also assigned to Cluster C4. Only five defects from this class are assigned to Cluster C5. The value of the silhouette index is 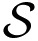 = 0.348.

### 4.3. Unsupervised Classification with DTW

Two different unsupervised classification methods based on the normalized DTW distance have been developed and tested. The first one directly applies the *K*-medoids algorithm over the defect set, while the second one applies the *K*-medoids algorithm to the prototypes obtained by the ESOM.

#### 4.3.1. Results with DTW and *K*-Medoids

The pairwise DTW distance between all of the defects in the dataset has been computed, and the *K*-medoids algorithm was applied to discover the underlying defect classes.

To find out the number of clusters, different values of *K* ∈ {2,…, 10} have been tried out. The *K* value with the highest global silhouette value is selected as the number of defect classes. Then, the *K*-medoids algorithm is applied. This clustering algorithm is sensitive to the initialization, which is performed by randomly choosing *K* defect classes. To minimize this effect, the algorithm is executed 100-times for every *K* value.

**Figure 6:**
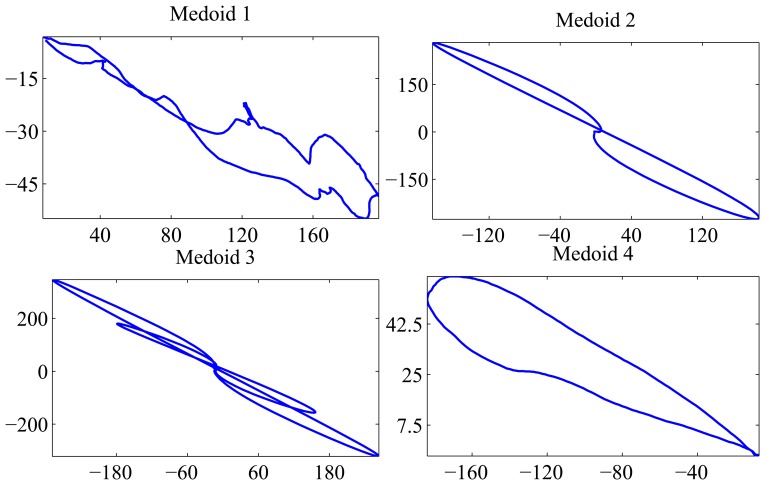
Resultant medoids represented in the complex impedance plane.

Four classes are found, and their medoids are depicted in Figure 6. Medoid 1 has only one lobe spreading across the right side of the complex impedance plane. Medoid 2 has two lobes in the first and third quadrant. Medoid 3 exhibits more than two lobes in the same quadrants. Finally, Medoid 4 has only one lobe in the left side of the impedance plane. The medoid shapes agree with the representative elements of each defect class shown in Figure 2. The global silhouette index is 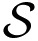 = 0.597.

**Figure 7:**
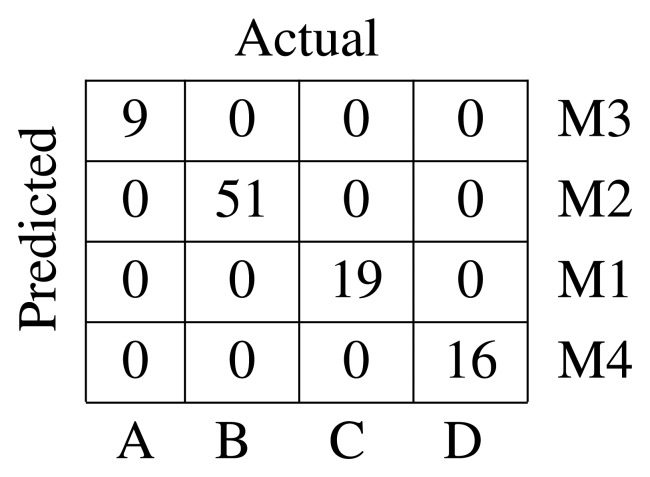
Confusion matrix for DTW-based clustering.

The labels assigned by the clustering algorithm are arbitrary. Looking at the resulting medoids, it is evident that Medoid 1 correspond to Class C, Medoid 2 to Class B, Medoid 3 to Class A and Medoid 4 to Class D. It is possible to rearrange the label names and compute a confusion matrix. The confusion matrix is shown in Figure 7. It can be seen that the unsupervised classifier is capable of gathering in the same cluster all pf the defects belonging to the same class with no error.

#### 4.3.2. Results with DTW and ESOM

**Figure 8:**
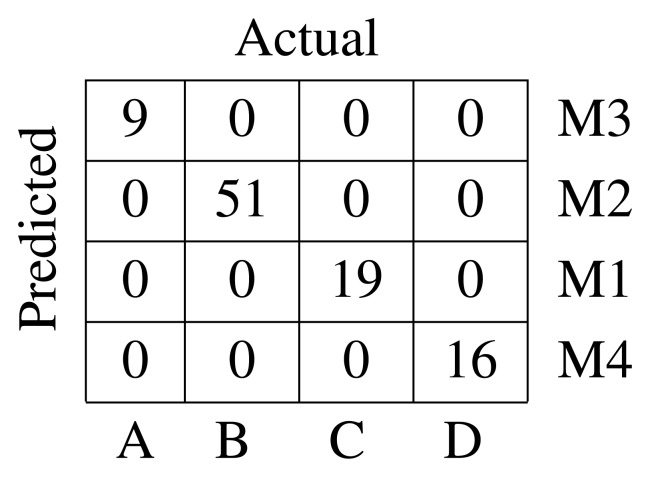
Confusion matrix for DTW and ESOM-based clustering.

In this final case, the *K*-medoids algorithm is applied to the prototypes obtained by the ESOM. The parameters used in the ESOM were *σ* = *ϵ* = 0.025, *γ* = 0.01, and *τ* = 10. They were empirically found. As before, the number of clusters is selected by maximizing the silhouette index. It is of importance to note that once the prototypes have been clustered, the defects are assigned to a cluster according to the closest prototype. The confusion matrix is shown in Figure 8, and the global silhouette index is 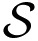 = 0.597.

Temperature influence has not been considered, because it did not change during experimentation, so the possible influence on system behavior could not be studied. The processing speed changes in a natural way during production. Since the wire is pulled by the forming coil, the speed increases linearly along time. Due to a constant sampling rate, the signal associated with a defect shrinks as speed increases. The DTW deals with this effect by nature provided the sampling rate is high enough to allow a precise shape reconstruction.

## 5. Discussion

**Figure 9:**
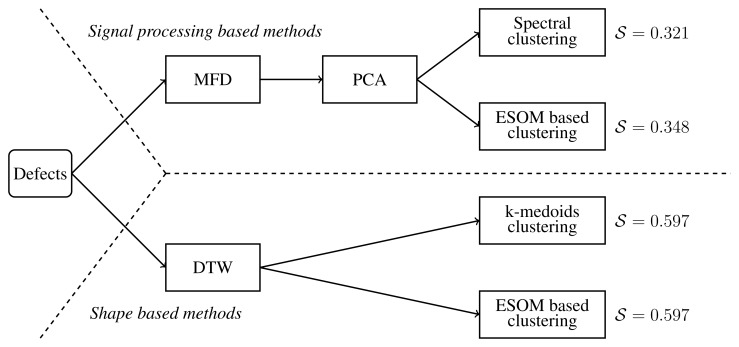
Algorithm description.

The results obtained show that both DTW-based methods, with ESOM and without ESOM processing, are capable of classifying the defects in an unsupervised fashion without error. Moreover, the methods that apply the normalized DTW distance outperform the MFD-based methods for the problem of classifying surface defects in the wire rod obtained by a eddy current inspection system. A schematic representation of the methods used is shown in Figure 9. The ESOM is very sensitive to the value given to *ϵ*. For instance, if *ϵ* = 0.035, the algorithm merges Classes C and D. The reason is that the *ϵ* parameter controls the number of prototypes and the distances between them. If *ϵ* is large, the number of prototypes is small and the distance is large. Hence, two clusters can merge into one. Since the class population is unbalanced, a small value of *ϵ* is needed to ensure that every class has enough prototypes. For *ϵ* = 0.025, the number of prototypes is 85, which is close to the total number of defects.

The inclusion of the ESOM processing in the DTW-based clustering algorithm presents an important advantage. ESOM is an on-line learning method, which is able to adapt the prototypes each time that a new defect is processed. Thus, a parsimonious representation of the historical surface defects is encoded in the ESOM network by a number of prototypes and their connections. Hence, a large database of defects need not be stored.

## 6. Conclusions

An efficient new unsupervised method for classifying surface defects in wire rod manufacturing has been developed. The defects are obtained by an eddy current inspection system. The new method is based on the DTW distance, which is used to measure the dissimilarity between the defects and uses an evolving self-organizing map to obtain a representative set of defect prototypes for each production shift. These prototypes are later classified using a *K*-medoids clustering algorithm.

The performance of the new method was demonstrated using a collection of real defects obtained in a manufacturing plant. This collection of defects was labeled by experts. The proposed method outperforms the classification methods based on modified Fourier descriptors that have also been applied to classify eddy current signals.

The developed method was conceived of as a computer tool to be applied offline, after a production shift, and to help the plant operators to automatically discover and classify the possible surface defects in the manufactured product. The DTW properties allows the method to deal with possible changes in production speed and the different sizes of defects belonging to the same class.
